# Biofunctional response of a synthetic ceramic of 99.9% tricalcium phosphate associated with a heterologous fibrin biopolymer and infrared photobiomodulation

**DOI:** 10.3389/fbioe.2025.1748343

**Published:** 2026-01-12

**Authors:** Carlos Henrique Bertoni Reis, Brenda Thaynne Lima de Matos, Cleuber Rodrigo de Souza Bueno, Benedito Barraviera, Rui Seabra Ferreira Júnior, Paulo Sérgio da Silva Santos, Marco Antonio Hungaro Duarte, Murilo Priori Alcalde, Dayane Maria Braz Nogueira, Geraldo Marco Rosa Júnior, Daniela Vieira Buchaim, Rogério Leone Buchaim

**Affiliations:** 1 Department of Biological Sciences, Bauru School of Dentistry, University of São Paulo, Bauru, Brazil; 2 Beneficent Hospital (HBU), University of Marilia (UNIMAR), Marilia, Brazil; 3 Department of Anatomy, Medical and Dentistry School, University Center of Adamantina (FAI), Adamantina, Brazil; 4 Department of Anatomy, Dentistry School, Faculty of the Midwest Paulista (FACOP), Piratininga, Brazil; 5 Center for the Study of Venoms and Venomous Animals (CEVAP), São Paulo State University (Univ Estadual Paulista, UNESP), Botucatu, Brazil; 6 Medical School, São Paulo State University (Univ Estadual Paulista, UNESP), Botucatu, Brazil; 7 CTS-CEVAP (Center for Translational Science and Biopharmaceutical Development), São Paulo State University (Univ Estadual Paulista, UNESP), Botucatu, Brazil; 8 Department of Surgery, Stomatology, Pathology and Radiology, Bauru School of Dentistry, University of São Paulo (USP), Bauru, Brazil; 9 Department of Dentistry, Endodontics and Dental Materials, Bauru School of Dentistry, University of São Paulo (FOB/USP), Bauru, Brazil; 10 Postgraduate Program in Applied Dental Sciences, Bauru School of Dentistry (FOB/USP), University of Sao Paulo, Bauru, Brazil; 11 Graduate Program in Anatomy of Domestic and Wild Animals, Faculty of Veterinary Medicine and Animal Science, University of São Paulo (FMVZ-USP), São Paulo, Brazil

**Keywords:** biocompatible materials, bone regeneration, bone repair, fibrin biopolymer, fibrin glue, fibrin sealant, low-level laser therapy, photobiomodulation

## Abstract

Bioproducts and biomaterials for repairing large bone defects hold significant promise in translational research, particularly within Medicine and Dentistry. This study investigated a novel biocomplex comprising a synthetic tricalcium phosphate biomaterial (B), a heterologous fibrin biopolymer formulation (F), and an intraoperative photobiomodulation (PBM) protocol to enhance critical-sized bone defect repair in rats. Sixty male Wistar rats were randomly allocated to six groups (*n* = 10 per group): CG (blood clot control), PCG (PBM + clot), FG (fibrin), PFG (PBM + fibrin), BFG (biomaterial + fibrin), and PBFG (PBM + biomaterial + fibrin). An 8.0 mm critical-sized defect was surgically created in the parietal bone of each animal; groups PCG, PFG, and PBFG received PBM using an 808 nm infrared laser at 100 mW output power intraoperatively. Animals were euthanized at 14 and 42 days post-surgery, followed by assessments of bone repair using micro-CT, histomorphological and morphometric analyses, and immunohistochemistry. Micro-CT analyses showed progressive defect repair across all groups, with notable closure in CG (clot alone) and PFG (PBM + fibrin). Biomaterial particles in BFG and PBFG obscured radiographic visualization of new bone formation. At 14 days, significant differences emerged between CG and both PFG and PBFG (*p* < 0.05), with no other intergroup differences. By 42 days, CG exhibited significant differences from PFG and PBFG (*p* < 0.05), alongside differences between PFG and BFG, and BFG and PBFG; remaining comparisons were non-significant. Immunohistochemical markers of bone remodeling were present in all groups, indicating active repair processes. In conclusion, the combination of fibrin biopolymer and PBM proved effective in promoting bone repair and neogenesis in critical calvarial defects.

## 1 Introduction

The management of large bone defects has still been a challenging problem for medical and dental specialties due to the complexity of available treatments, significant morbidity and high incidence of late complications (Kamal et al., 2019; Kurowiak et al., 2025). Combined with an increasing prevalence of trauma, congenital anomalies and degenerative diseases that can compromise the restoration of bone architecture, tissue engineering and regenerative medicine seek to develop reconstructive therapies to regenerate lost bone and restore its function (Cunha F. B. et al., 2021; Santoro et al., 2025). Among the various reconstructive methods, autogenous grafting provides the most favorable repair conditions, as it combines all the characteristics required for bone regeneration in terms of osteoconduction, osteoinduction and osteogenesis (McAllister and Haghighat, 2007; Yoshikawa et al., 2009; Inchingolo et al., 2022).

However, there are several factors that limit their application, including the risk of morbidity at the donor site and limited tissue availability, which allows advances in the development and improvement of new biomaterials (Bigham-sadegh and Oryan, 2015; McGovern et al., 2018; Schindeler et al., 2018). In view of this, in recent decades, bone substitutes have been the subject of intense research, with the aim of overcoming the limitations resulting from graft harvesting or the use of bone banks, and thus assisting and accelerating the regenerative process, repairing the lesion with new tissue with native morphofunctional characteristics (Fernández-Bodereau et al., 2019; Steijvers et al., 2022; Ferraz, 2023).

Given the wide diversity of commercially available biomaterials, previous studies have presented scientific evidence and predictability of clinical success in the use of synthetic ceramics (Shi et al., 2018; Latimer et al., 2021; De Pace et al., 2025; Duarte et al., 2025; Rajasekar et al., 2025). The biomaterial QualyBone TCP (β-TCP; QualyLive^®^, Amadora, Portugal) is a synthetic ceramic, containing 99.9% tricalcium phosphate, whose main objective is to fill bone defects or cavities, in the search for bone regeneration and growth, stimulating the proliferation and differentiation of osteoblasts. In addition, there is no immunological or infection risk, it is a radiopaque material and easy to manipulate clinically, a fact that allows for a reduction in surgery time (Reis et al., 2022; Mahesh Saurabh Juneja, 2024).

Thus, the physicochemical properties of these ceramics give the biomaterial remarkable characteristics for providing biomechanical support to cells, ensuring bone growth (Šponer et al., 2011; Vaiani et al., 2023). Although this method is clinically established, a new therapeutic approach for bone regeneration is currently being employed with the use of specialized tissue constructions to achieve a synergistic effect and better overall properties when compared to conventional grafting techniques (Borie et al., 2015; Mendoza-Cerezo et al., 2023).

Among the tissue engineering constructions for bone repair, the association of three-dimensional scaffolds is based on the attempt to mimic the native bone microstructure, facilitating the recruitment of osteogenic cells, growth factors *in situ* and promoting the synthesis of new mineralized bone matrix (Kim et al., 2014; Zhu et al., 2019). In this context, natural biopolymers, such as fibrin sealants, have become ideal candidates for use in combination with particulate bone grafts (Khalili et al., 2024). This is because it enables the manufacture of multifunctional scaffolds that stop bleeding through homeostatic mechanisms, increase resistance to shear stress, graft stability in the surgical bed, a preponderant factor in preventing micromotion, and provide longer cellular support during the entire bone repair process, increasing the graft success rate (Kolehmainen and Willerth, 2012; de Oliveira et al., 2020).

The fibrin sealant precursors, fibrinogen and thrombin, interact in the final stages of the blood coagulation cascade resulting in a cross-linked fibrin matrix, a temporary structure necessary to support healing and tissue remodeling (Zubairova et al., 2015). In addition, fibrin specifically binds to numerous proteins and growth factors released in response to injury, through interactions with specific cell surface receptors, a preponderant factor to play an active role in the repair process (Noori et al., 2017; Li et al., 2024). Most preparations consist of plasma blood components, which allows them to be classified according to the method of obtaining fibrinogen, in autologous or homologous fibrin sealants. However, autologous formulations become unviable in seriously injured patients or in unforeseen emergencies, and homologous formulations with high added value and risk of viral transmission (Taniguchi et al., 2022).

The identification of these methodological limitations prompted the team of researchers at the Center for the Study of Venoms and Venomous Animals (CEVAP, Sao Paulo State University UNESP, Botucatu, Brazil) to develop a modified version of these preparations as an effective, safe and affordable alternative. Thus, human fibrinogen was replaced by plasma fibrinogen from large animals, *Bubalus bubalis*, and thrombin by serine protease, extracted from the venom, *Crotalus durissus terrificus* (Ferreira et al., 2017). Initially, the protein concentrations of serine protease and heterologous cryoprecipitate were designed for the treatment of chronic venous ulcers and peripheral nerve repair, as an alternative to conventional sutures, presenting satisfactory preclinical and clinical results (Buchaim et al., 2015; Biscola et al., 2017). In fact, the excellent biocompatibility, controllable biodegradability, intrinsic bioactivity and many other unique characteristics make this therapeutic formulation viable and attractive for other areas such as tissue bioengineering and regenerative medicine (Song et al., 2018). Thus, improvements in research and the use of new technologies have directed the applicability of heterologous fibrin sealant as a three-dimensional scaffold in bone reconstruction, a delivery system for biologically active molecules and support for mesenchymal stem cells, which led to the change of name to heterologous fibrin biopolymer (Barros et al., 2009; Machado et al., 2015; Buchaim et al., 2019).

In the search for improved results in reconstructive surgical interventions that require tissue repair, several extraoperative therapeutic modalities have been researched. Among non-invasive treatments, laser photobiomodulation (PBM) has been widely used in several clinical conditions in order to accelerate tissue regeneration and modulate inflammatory processes in cells with functional deficits (Pinheiro and Gerbi, 2006; De Freitas and Hamblin, 2016; Hamblin, 2016; de Freitas Dutra Júnior et al., 2022). Not unlike what occurs in bone tissue, PBM has been shown to be effective in modulating biochemical reactions, increasing the supply of adenosine triphosphate (ATP), cell membrane permeability, enabling calcium influx, stimulating cell differentiation and proliferation, regulating growth factors and pro-inflammatory cytokines, inducing collagen synthesis and remodeling, and angiogenesis (Nejatifard et al., 2021).

This sum of cellular events stimulated by PBM causes the injured bone tissue to reestablish its homeostasis, that is, the normalization of its shape and function, leading to morphofunctional regeneration (Kazancioglu et al., 2013; Rosso et al., 2017; Dias et al., 2023; Rodrigo et al., 2023). Our team has employed a PBM protocol with satisfactory results, but which requires several applications. In addition, there is a need to reformulate the concentrations of the fibrin biopolymer blood components to achieve a less dense three-dimensional mesh, which provides a microenvironment more conducive to cell migration, to achieve ideal characteristics such as a scaffold, providing agglutination of the particulate graft and preventing invagination of surrounding soft tissues, promoting guided tissue regeneration without the use of membranes.

Despite extensive investigation of photobiomodulation (PBM) for bone repair, current evidence remains fragmented, with heterogeneous wavelengths, doses, and application schedules leading to inconsistent outcomes and preventing robust dose–response generalizations or clinical standardization. Likewise, β-tricalcium phosphate (β-TCP) ceramics are widely recognized as osteoconductive, resorbable bone substitutes, yet their integration with advanced bioactive scaffolds is still being optimized to improve defect filling and long-term biomechanical performance. In parallel, a heterologous fibrin biopolymer derived from buffalo cryoprecipitate and snake venom serine protease has emerged as a versatile hemostatic, adhesive, and scaffold material, with growing experimental and clinical evidence supporting its application in nerve and bone regeneration, but with limited data on formulations with reduced fibrinogen content in critical-sized bone defects. Therefore, there is a compelling need to investigate whether a single-session intraoperative PBM protocol, applied at a well-defined infrared dose, can act synergistically with a 99.9% β-TCP ceramic and a low-fibrinogen heterologous fibrin biopolymer to enhance early bone regeneration in critical calvarial defects, potentially informing more standardized and translatable regenerative strategies.

## 2 Materials and methods

### 2.1 Ethical aspects

The research project was approved by the Animal Use Ethics Committee (CEUA) of the Bauru School of Dentistry–University of São Paulo (FOB-USP) Protocol 005/2020 dated 1/14/2021 (amendment 9/19/2023). The sample size was determined *a priori* following the 3Rs principle (Replacement, Reduction, Refinement), prioritizing the minimum number of animals necessary to achieve reliable, statistically significant results while maintaining data integrity (Cornwell, 2015; MacArthur Clark, 2018). This approach was informed by previous experiments conducted by our research group using comparable critical-sized calvarial defect models and treatment protocols, which consistently demonstrated robust differences in new bone formation between treatment and control groups using cohorts of 5–10 animals per group/time point (Pomini et al., 2022; Rossi et al., 2024). Based on these precedents, which yielded effect sizes typically exceeding d = 1.0 for key comparisons and ANOVA F-values >5.0 (*p* < 0.01), we determined that *n* = 10 animals per group (5 per time point) provided adequate statistical power (>80%) to detect meaningful treatment effects at *α* = 0.05, thereby avoiding excess animal use while ensuring experimental validity (Buchaim et al., 2022).

### 2.2 Experimental design

Sixty adults male Wistar Hannover rats (*Rattus norvegicus*), aged 90 days and weighing approximately 330 g, were used. They were obtained from the Bioterium of the University of Sao Paulo - USP (Ribeirao Preto, Brazil) and kept in the Bioterium of the Bauru School of Dentistry (FOB/USP, Bauru, Brazil). The animals were kept in conventional cages with rodent food and filtered water “*ad libitum*”, in air-conditioned environment with exhaust fan, controlled light-dark period of 12 h each, average temperature of 22 °C, and all provisions for monitoring the animals were followed.

**FIGURE 1:**
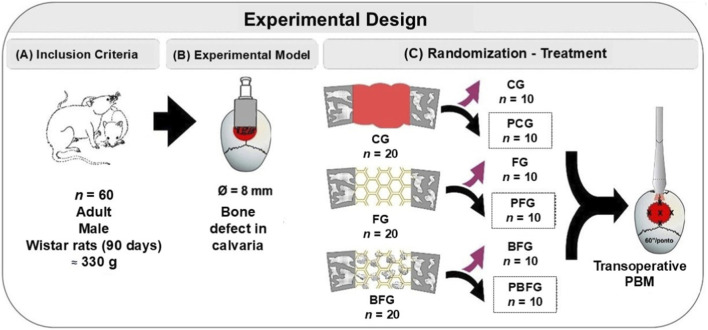
Experimental design schematic. **(A)** Inclusion criteria: 60 male Wistar rats (90 days, ∼330 g). **(B)** 8 mm critical-sized calvarial defect model. **(C)** Six experimental groups (*n* = 10): CG (clot), PCG (PBM + clot), FG (fibrin), PFG (PBM + fibrin), BFG (β-TCP + fibrin), PBFG (PBM+β-TCP + fibrin). PBM applied at five points (808 nm, 100 mW, single session).

The rats were randomly divided into six groups according to the type of defect filling and photobiomodulation treatment: CG (Clot group); PCG (PBM + Clot); FG (Fibrin group); PFG (PBM + FG); BFG (Biomaterial group + Fibrin); PBFG (PBM + Biomaterial + F) (Figure 1).

### 2.3 Treatments

#### 2.3.1 Tricalcium phosphate ceramic (β-TCP)

QualyBone TCP (QualyLive^®^, Amadora, Portugal) is a synthetic porous ceramic primarily designed for filling bone defects or cavities. It consists of 99.9% tricalcium phosphate and holds the Conformité Européenne (CE0120) certification, or “European Conformity”, a mandatory seal for any manufacturer, European or otherwise, wishing to sell products within European territory. This certification ensures compliance with a defined set of parameters aligned with European Union standards. Additionally, it meets the ISO 13485:2016 certification requirements for the design and manufacture of sterile synthetic bone substitutes. The granulation used is medium, ranging from 0.5 to 1.0 mm.

#### 2.3.2 Heterologous fibrin biopolymer

The heterologous fibrin biopolymer, previously called fibrin sealant derived from snake venom, was provided by the Center for the Study of Venoms and Venomous Animals at São Paulo State University (CEVAP, UNESP/Botucatu, Brazil). The composition and application method are thoroughly described in patent BR 102014011432-7, granted on 6 July 2022, by the Brazilian National Institute of Industrial Property (INPI).

The biopolymer consists of three distinct solutions that are thawed, combined, and homogenized before application. The first solution exhibits thrombin-like properties (gyroxin) and is derived from the venom of *Crotalus durissis terrificus*, with calcium chloride serving as the diluent. The second solution contains fibrinogen (cryoprecipitate), extracted from the blood of *Bubalus bubalis*, the buffalo.

In this study, we used the protocol by Pomini et al. (2022) (Pomini et al., 2023) with equal proportions of the three components (20 µL of each), presenting a reduced concentration of fibrinogen, in relation to the initial studies with the fibrin biopolymer in bone regeneration (Vigliar et al., 2024).

#### 2.3.3 Laser photobiomodulation therapy protocol

The PCG, PFG, and PBFG groups underwent low-level laser therapy treatment with the Therapy EC equipment (DMC^®^, São Carlos, Brazil). The equipment allows the application of infrared laser at a single wavelength of 808 nm. The Therapy EC used in this PBM protocol is registered with the Brazilian Health Surveillance Agency (Anvisa) under number 80030819013, serial number 48555, manufactured in 03/2022, batch 0041. The samples were irradiated directly onto the skin of the surgical area, without the use of optical fibers, with a distance between the laser tip and the bone tissue of the calvaria of approximately 2 mm.

The laser irradiation was performed in continuous-wave mode in the infrared spectrum, using gallium–aluminum–arsenide (GaAlAs) as the active medium. The beam area of the device, according to the manufacturer, was 0.0984 cm^2^, with an output power of 100 mW, resulting in an irradiance of 1.016 W/cm^2^ at the target. The energy density was 60.98 J/cm^2^ per point, with an exposure time of 60 s per point. The irradiation was applied at four sites on the defect surface in a clockwise pattern (12, 3, 6, and 9 o’clock), as well as at a central point, in a single session. Each point received an energy dose of 6 J, totaling 30 J across the entire treated area. Only one application was performed in the immediate postoperative period, after performing the surgical suture, on the skin in the area (de Freitas et al., 2018; Pomini et al., 2023).

The dosimetry calculation describes laser therapy parameters: output power P=100 mW (equivalent to 0.1 W), beam area A=0.0984 cm^2^, irradiance I=1.016 W/cm^2^, and exposure time t=60 s per point. Energy delivered to each point is calculated as E=P×t . Substituting values: E=0.1 W ×60 s =6 J. This represents the total energy deposited at the treatment site. Fluence, or energy density, is $F=\frac{E}{A}$ . Thus, $F=\frac{6\;J}{0.0984{\;\text{cm}}^{2}}=60.98$ J/cm^2^, quantifying energy per unit area for tissue interaction. Irradiance is $I=\frac{P}{A}=\frac{0.1\;W}{0.0984{\;\text{cm}}^{2}}=1.016$ W/cm^2^, confirming power density at the target and consistency across parameters.

### 2.4 Experimental surgery

The surgical procedures were conducted in the surgery laboratory of the Bioterium at the Bauru School of Dentistry (FOB-USP). During the experimental surgery, the animals underwent general intraperitoneal anesthesia in the lower left abdominal quadrant. This was achieved through a combination of the sedative ketamine hydrochloride (80 mg/kg of body weight; Dopalen^®^, Sespo Indústria e Comércio Ltda, SP, Brazil) and the muscle relaxant xylazine hydrochloride (10 mg/kg of body weight; Anasedan^®^, Sespo Indústria e Comércio Ltda, SP, Brazil), under strict monitoring.

Following anesthesia, trichotomy was performed using a hair trimmer (Philips^®^ Multigroom QG3250, SP, Brazil) in the frontal-parietal bone region, between the external ear pavilions. The specimens were then weighed using an analytical scale (MicroNal^®^ Precision Equipment, SP, Brazil). Antisepsis of the shaved region, including the surrounding fur, was carried out using a 10% topical solution of Polyvinyl Pyrrolidone Iodine PVPI (Povidine^®^ Antisséptico, Vic Pharma, Brazil). The surgical procedure was conducted independently on a sterile-covered bench, with new materials used for each specimen.

The animals were secured to the operating table in the ventral decubitus position. A 4 cm semilunar incision was then made using a No. 15 carbon steel scalpel blade (Embramac^®^, Campinas, Brazil) in the tegument. The periosteum was meticulously detached with a syndesmotome and reflected along with adjacent tissues, exposing the external surface of the parietal bones. A circular osteotomy, 8.0 mm in diameter, was performed at the center of the parietal bones using a trephine drill (Neodent^®^, Curitiba, Brazil) attached to a contra-angle handpiece (Driller^®^, Carapicuíba, Brazil) and powered by an electric micromotor (Driller^®^ BLM 600 Baby, Carapicuíba, Brazil). The procedure was conducted at low speed (1,500 rpm) under continuous and abundant irrigation with sterile saline solution (0.9%) to prevent thermal-induced bone necrosis. This technique ensured the removal of a smooth, rounded bone fragment without spicules, while preserving the integrity of the dura mater and brain.

In the CG and PCG groups, the defects were created but remained unfilled, allowing only clot formation. In the FG and PFG groups, the defects were treated with CEVAP fibrin biopolymer. Meanwhile, in the BFG and PBFG groups, the defects were filled with tricalcium phosphate ceramic integrated into the CEVAP fibrin biopolymer. Prior to application, the biomaterial was precisely weighed on an analytical balance (Micronal^®^ Precision Equipment, São Paulo, Brazil) to ensure complete filling of the surgical cavity (0.08 g).

After complete polymerization of the biopolymer with the ceramic, the resulting compound was carefully transferred to the defect site without exerting pressure on the brain. Following this step, all experimental groups were subdivided based on whether they received PBM treatment. The surgical area tissues were repositioned to ensure that the periosteum adequately covered the cavities. The integument was then sutured using simple stitches with 4-0 silk thread (Ethicon^®^, Johnson & Johnson, São Paulo, Brazil). The area was meticulously cleaned with gauze moistened in a topical antiseptic solution containing 2% chlorhexidine (Riohex^®^, Rioquímica, São José do Rio Preto, Brazil).

In the PCG, PFG, and PBFG groups, the laser was applied perpendicularly at four distinct points on the defect surface in a clockwise manner (12 o’clock, 3 o’clock, 6 o’clock, and 9 o’clock), along with an additional central point, in a single session. The animals were placed in lateral decubitus within their cages and exposed to incandescent light to facilitate full anesthetic recovery. Immediately after the surgical procedure, they received a single dose of the antibiotic Flotril^®^ 2.5% (Schering-Plough, Rio de Janeiro, Brazil) at 0.2 mL/kg and the analgesic Dipirona Analgex V^®^ (Agener União, São Paulo, Brazil) at 0.06 mL/kg *via* intramuscular injection. The analgesic regimen continued for 3 days, supplemented by acetaminophen (Paracetamol, Generic medication, Medley, São Paulo, Brazil) at 200 mg/kg, with six drops per animal dissolved in the drinking water until euthanasia.

Throughout the experiment, the animals were monitored for clinical signs of pain by assessing changes in behavior, including apathy, depression, aggression, or hyperexcitability—key indicators deviating from their usual demeanor. Observations also included alterations in gait, posture, and facial expressions. Additionally, food and water intake, physical condition, clinical symptoms, and overall behavior were systematically evaluated.

### 2.5 Surgical procedure for tissue collection

At 14- and 42-day post-surgery, five animals from each group were weighed and euthanized according to the general anesthetic overdose method, administering a triple dose (240 mg/kg ketamine + 30 mg/kg xylazine). Once death was confirmed, the defect region of each specimen was carefully extracted, ensuring the preservation of supraperiosteal soft tissues. The samples were then fixed in a 10% formalin solution with phosphate buffer (pH 7.2) for 48 h before being sent for computed microtomography analysis.

### 2.6 X-ray micro-computed tomography (micro-CT)

Following fixation in formalin, the specimens underwent X-ray beam scanning using a SkyScan 1174v2 computed microtomography system (Bruker-microCT^®^, Kontich, Belgium) at the Bauru School of Dentistry (Endodontics). The Cone-Beam X-ray sources were operated at 50 kV and 800 μA, utilizing a Cu + Al filter. Each specimen was carefully placed in tubes, positioned, and securely fixed within the appropriate sample holder using utility wax to ensure stabilization and prevent movement during scanning.

The samples were rotated 360° with a rotation step of 0.5, achieving an isotropic resolution of 19.6 µm, with an acquisition time of 41 min and 32 s per specimen. The resulting images were processed and reconstructed using specialized software, including 64Bits270013 (Bruker^®^, Kontich, Belgium) and NRecon^®^ (version 1.6.8.0, SkyScan Bruker-microCT), generating approximately 1,000–1,100 slices based on the defined anatomical parameters. Two-dimensional and three-dimensional visualization was conducted using Data Viewer^®^ (version 1.4.4, 64-bit) for linear measurements of coronal, transaxial, and sagittal axes, along with CTvox^®^ (version 2.4.0 r868, Bruker Micro CT) for enhanced image analysis, facilitating qualitative assessment of newly formed bone tissue.

### 2.7 Histotechnical processing

After acquiring the microtomographic images, the specimens were rinsed in running water for 24 h and then subjected to demineralization using an ethylenediaminetetraacetic acid (EDTA) solution. This solution contained 4.13% Tritiplex^®^ III (Merck KGaA, Hessen, Germany) and 0.44% sodium hydroxide (Labsynth, São Paulo, Brazil), with weekly solution changes over approximately 42 days. During these intervals, radiographic analyses were conducted using Insight adult IP-21 F-Speed periapical film (Carestream^®^ Carestream Health, New York, United States) to verify the progression of demineralization.

Once complete demineralization was confirmed, the specimens underwent dehydration through a graded series of ethyl alcohol concentrations, followed by diaphanization in xylene and embedding in Histosec^®^ paraffin (Merck, Hessen, Germany). Subsequently, semi-serial coronal sections were prepared, focusing on the central defect region, using a Leica^®^ RM2245 semi-automatic microtome (Leica Biosystems^®^, Wetzlar, Germany). The sections were cut at a thickness of 5 µm for subsequent staining with hematoxylin-eosin (HE), Masson’s trichrome (MT), Picrosirius-red (PRS), and immunostaining procedures.

### 2.8 Histomorphologic and histomorphometric analysis

For the histomorphological assessment of bone defect areas, the entire extent of the defect was considered in all specimens to analyze the bone repair pattern across all groups. This allowed for the evaluation of granulation tissue presence, inflammatory infiltrate, the formation and quality of immature or mature/lamellar bone, and the degree of newly formed tissue filling.

To achieve this, four semi-serial sections of the surgical bed from each defect were examined under an Olympus^®^ BX50 light microscope (Olympus Corporation, Tokyo, Japan), with images captured using ×4 and ×20 objectives and a digital camera (Olympus DP 71^®^, Tokyo, Japan). The imaging process was conducted using the DP Controller^®^ 3.2.1.276 software (2001–2006, Olympus Corporation, Tokyo, Japan), configured to a resolution of 4,080 × 3,072 pixels and 30% spot size, in the Anatomy Research Laboratory of the Bauru School of Dentistry (FOB-USP).

Volume density (VVi) represents the fraction of volume occupied by specific components—such as graft material, inflammatory infiltrate, connective tissue, bone tissue, and bone marrow—within the total defect site (graft + reactive tissue). In histological sections, this is expressed as an area fraction, defined by VVi = AAi. The volume density assessment followed a structured protocol. Images encompassing the entire defect were captured using a ×4 objective and saved in TIFF format. The defect was then reconstructed in Adobe Photoshop CS6. Subsequently, the images were analyzed in AxioVision^®^ software (version 4.8, Carl Zeiss, Jena, Germany), where the total analyzed area (A) and the area occupied by each constituent within the defect (Ai) were measured using pixel-based quantification. The volume density (VVi) of each structural component was determined by the equation: VVi = AAi = Ai/A × 100 (Weibel, 1969).

### 2.9 Birefringence analysis of collagen content of bone defects

Picrosirius-red stained sections were examined under polarized light to assess the quality and quantity of the newly formed organic matrix throughout the defect healing periods. Defect images were captured using a Leica DFC 310FX high-resolution digital camera (Leica^®^, Microsystems, Wetzlar, Germany) connected to a Leica DM IRBE confocal laser microscope and LAS 4.0.0 image acquisition system (Leica^®^, Microsystems, Heerbrugg, Switzerland) at the Centro Integrado de Pesquisas (CIP, FOB-USP).

To evaluate collagen quality based on the birefringence of fiber bundle organization, the central defect fields were analyzed under a polarized light microscope at ×10 magnification. Three histological fields, representing the entire defect extension, were imaged. To prevent interference in fiber quantification, all residual bone within these fields were removed using Adobe Photoshop CS6 software.

The images were then processed in AxioVision^®^ imaging software (Carl Zeiss MicroImaging GmbH, Jena, Deutschland). Through the interactive Processing-Segmentation-Threshold tool, the RGB color pattern was defined for each structure. Bone tissue was distinguished by its random and disorganized fibrillar arrangement, with polarization colors varying from red-orange (indicating poorly organized bone) to bright green/yellow (denoting lamellar bone), depending on fiber width (Bossini et al., 2012; Costa et al., 2019; Nogueira et al., 2022).

### 2.10 Immunohistochemical analysis

For immunohistochemical analysis, histological sections were deparaffinized in xylene and rehydrated through a graded ethanol series (100° - 100° - 100° - 90° - 70° GL). Antigen retrieval was carried out by immersing the histological slides in citrate buffer (Spring Bioscience) within a pressurized chamber (Decloaking Chamber^®^, Biocare Medical) at 95 °C for 20 min. Following each stage of the immunohistochemical reaction, the slides were rinsed in PBS 0.1 M, pH 7.4 (Sigma Aldrich^®^).

Next, the slides were incubated in 3% hydrogen peroxide for 1 h to block endogenous peroxidase, followed by immersion in 1% bovine serum albumin (Sigma Aldrich^®^) for 12 h to minimize nonspecific binding. Samples from each experimental group were divided into four batches, with each batch incubated with one of the following primary antibodies: BMP-2 Ab-AF5163 (Affinity Bioreagents^®^, Golden, CO, USA, batch 15v8605), VEGFA Ab-AF5131 (Affinity Bioreagents^®^, Golden, CO, USA, batch 63m6093), BMP-4 Ab-AF5175 (Affinity Bioreagents^®^, Golden, CO, USA, batch 60g7275), OSTEOCALCIN Ab-DF12303 (Affinity Bioreagents^®^, Golden, CO, USA, batch 85n2245), and ACP-5 Ab-DF6989 (Affinity Bioreagents^®^, Golden, CO, USA, batch 70b0275) (Nogueira et al., 2025).

The sections were subsequently treated with a biotinylated anti-mouse/rabbit IgG secondary antibody generated in horse (BA-1400, Vector Laboratories) for 2 h, followed by incubation with streptavidin conjugated to horseradish peroxidase (HRP) (SA-5004, Vector Laboratories^®^) for 1 h.

For visualization, the reaction was developed using 3,3′-diaminobenzidine tetrahydrochloride (SK-4105, Vector Laboratories^®^) as the chromogen. Counterstaining with Harris hematoxylin was applied to samples for VEGF, OCN, TRAP, BMP2, and BMP4 detection, enhancing immunostained cell visibility. The slides then underwent ethanol dehydration, diaphanization in xylene, and final mounting with coverslips and mounting medium (Fisher Scientific^®^).

As a negative control, the specimens underwent identical procedures but without primary antibody application. Immunohistochemical analysis was conducted by a blind researcher (CR), with the region of interest encompassing the full extent of the bone defect.

### 2.11 Statistical analysis

Data were analyzed using one-way ANOVA to assess differences among treatment groups at each time point. The assumptions of normality and homogeneity of variances were verified with the Shapiro–Wilk and Bartlett tests, respectively, at a 5% significance level. Upon finding a significant overall effect, effect size was estimated using eta-squared (η^2^), calculated as the ratio of the between-group sum of squares to the total sum of squares, to quantify the proportion of variance explained by treatment. Subsequently, *post hoc* mean comparisons were performed using Tukey’s test, and Cohen’s d was calculated for selected pairwise contrasts to determine the magnitude of differences in new bone formation between groups. Intragroup comparisons between 14 and 42 days were conducted using the Student’s t*-*test, with corresponding Cohen’s d values. All analyses were carried out in GraphPad Prism (version 8), adopting *p* < 0.05 as the threshold for statistical significance.

## 3 Results

No changes in the animals’ behavior were observed beyond the usual post-surgical pattern for critical calvarial bone defects. The medication was administered on the scheduled days, and no animal died before the planned euthanasia.

### 3.1 X-ray micro-computed tomography (micro-CT)

**FIGURE 2:**
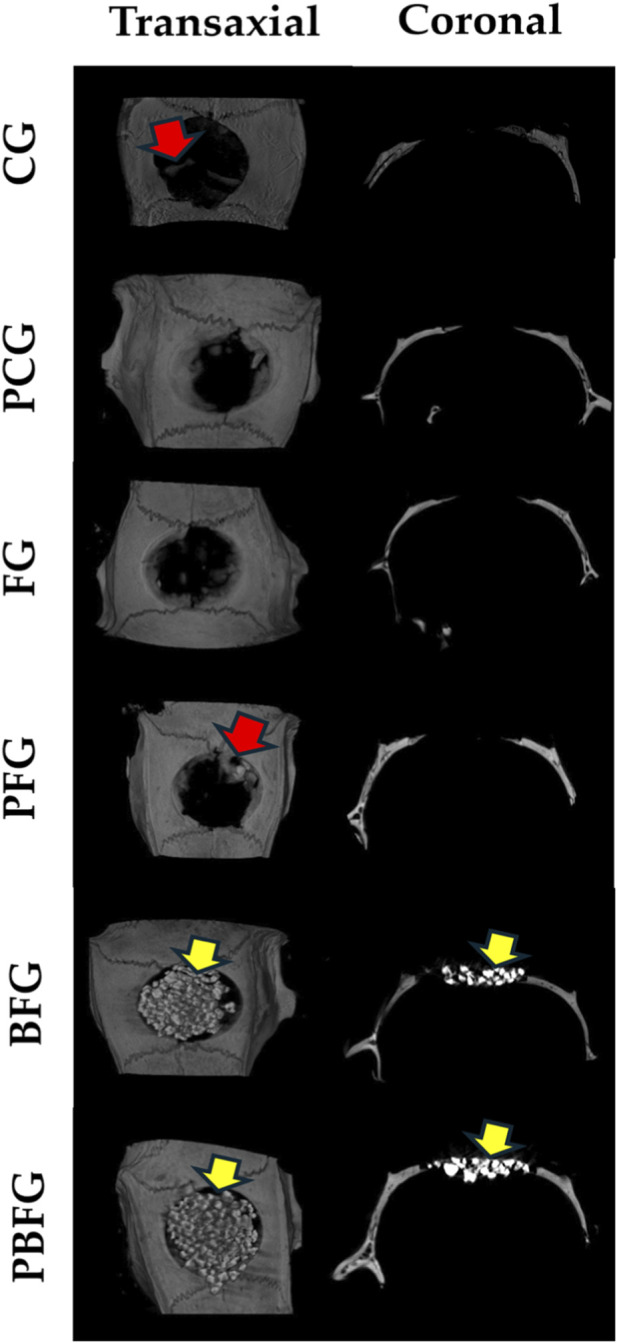
Micro-CT images (transaxial/coronal views) of 8 mm calvarial defects at 14 days post-surgery. CG (clot), PCG (PBM + clot), FG (fibrin), PFG (PBM + fibrin), BFG (β-TCP + fibrin), PBFG (PBM+β-TCP + fibrin). Yellow arrows: β-TCP particles. Red arrows: centripetal new bone formation.

At 14 days postoperatively, micro-CT images demonstrate the progression of defect repair, primarily in the clot-filled group (CG) and the group treated with fibrin biopolymer combined with low-power laser (PFG). Due to their radiopacity, biomaterial particles hinder the visualization of new bone formation in the BFG and PBFG groups. The hypodense cavity, corresponding to the critical bone defect, can be qualitatively observed, laterally delimited by the hyperdense bone border and irregular hyperdense regions associated with centripetal bone neoformation. Similarly, in the BFG and PBFG groups, visualization is challenging due to the presence of the hyperdense biomaterial (Figure 2).

**FIGURE 3:**
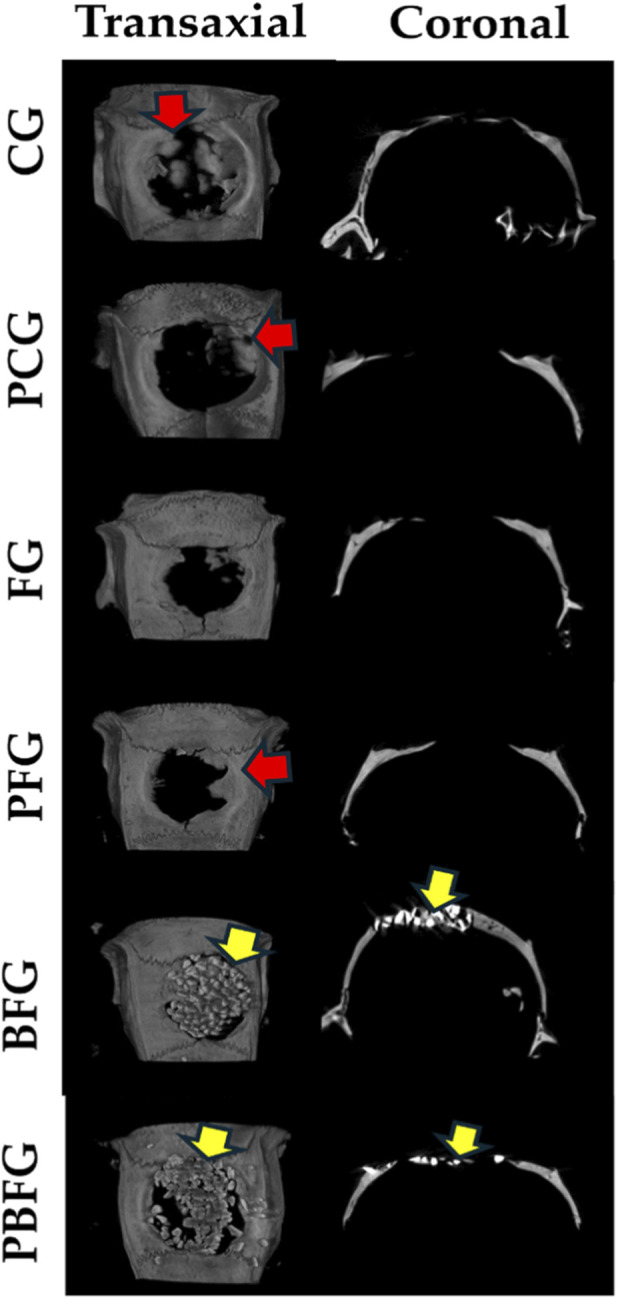
Micro-CT images (transaxial/coronal views) of 8 mm calvarial defects at 42 days post-surgery. CG (clot), PCG (PBM + clot), FG (fibrin), PFG (PBM + fibrin), BFG (β-TCP + fibrin), PBFG (PBM+β-TCP + fibrin). Yellow arrows: β-TCP particles. Red arrows: increased mineralization/new bone around remnants.

Microtomographic images of animals at the 42-day mark demonstrate a more advanced stage of defect repair, particularly in the clot-filled groups (CG) and the group treated with fibrin biopolymer combined with low-level laser (PFB), exhibiting a more organized structure compared to the 14-day micro-CT. Visually, distinguishing new bone formation from biomaterial particles in the BFG and PBFG groups remains challenging. However, due to the increased mineralization and radiopacity relative to the 14-day period, new bone formation can be observed around the biomaterial (Figure 3).

### 3.2 Histomorphological analysis (HE, MT and PRS)

The images demonstrate newly formed bone growth, with multiple gaps containing osteocytes, arranged in a disorganized, non-lamellar structure, and collagen fibers oriented in various directions. Greater evidence of bone growth and maturation is also observed near the defect’s edge, progressing in a centripetal manner.

**FIGURE 4:**
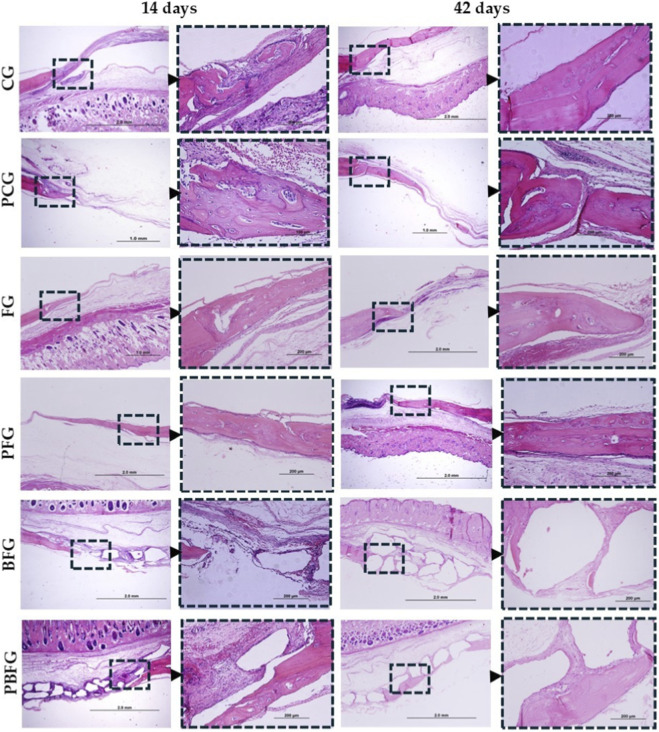
Hematoxylin-eosin (H&E) staining of 8 mm calvarial defects. 14 and 42 days post-surgery (4× left, 20× inset right). CG (clot), PCG (PBM + clot), FG (fibrin), PFG (PBM + fibrin), BFG (β-TCP + fibrin), PBFG (PBM+β-TCP + fibrin). Scale bars: 1–2 mm (4×), 200 μm (20×). Note enhanced neoformation in PBM groups and particle encapsulation (BFG/PBFG) at 42 days.

**FIGURE 5:**
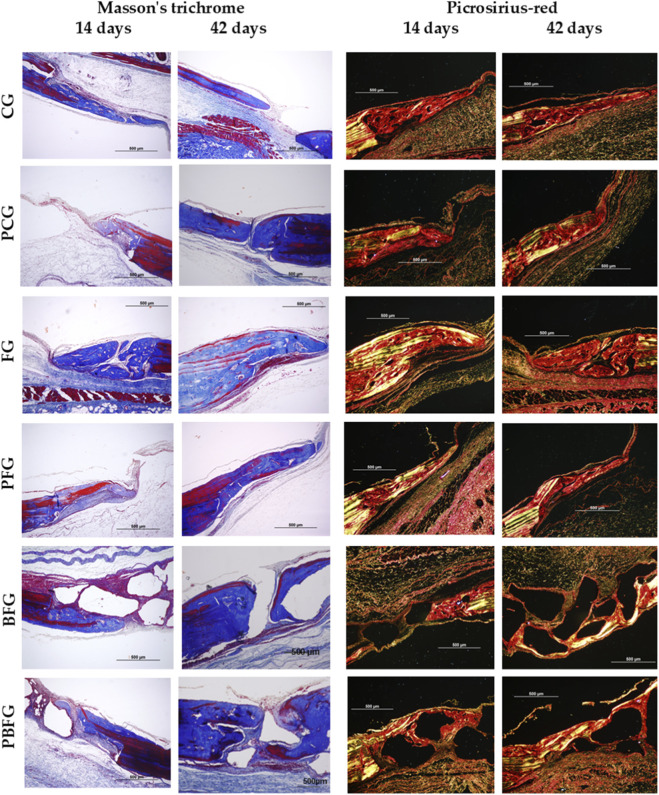
Masson’s trichrome (MT) and picrosirius red (PRS) staining under polarized light (20×) of 8 mm calvarial defects at 14/42 days. CG (clot), PCG (PBM + clot), FG (fibrin), PFG (PBM + fibrin), BFG (β-TCP + fibrin), PBFG (PBM+β-TCP + fibrin). Scale bar: 500 μm. PRS: red-orange (immature collagen, 14 days); yellow-green increase (lamellar bone, 42 days; prominent in PBM groups).

In both staining methods (HE and Masson’s Trichrome), during the 14-day period, the groups that exhibited the most significant new bone formation were PBFG, PFG, and PCG, all undergoing photobiomodulation. In groups containing biomaterial (BFG and PBFG), reactive tissue formation and small-scale bone neoformation were observed between biomaterial particles, following a centripetal pattern and appearing more prominently at the edges of the bone defect. In the groups where fibrin biopolymer was present alone (FG and PFG), it was observed within connective tissue as hyperchromatic corpuscles, alongside a discrete inflammatory infiltrate. The distinction between mature bone and primary bone tissue was evident in all groups (Figures 4, 5).

At 42 days, more advanced bone growth was observed, though the division between mature and immature bone was less distinct at the wound edge. The newly formed bone tissue exhibited greater organization, yet peripheral regions remained disorganized. In the biomaterial-containing groups (BFG and PBFG), centripetal bone formation around biomaterial particles was more pronounced, with some particles becoming completely encapsulated by bone tissue. In fibrin biopolymer-containing groups (FG, PFG, BFG, and PBFG), angiogenesis was more prominent than in other groups, particularly those subjected to photobiomodulation (PFG and PBFG) (Figures 4, 5).

The histochemical evaluation of collagen fibers enables the study of the structural arrangement and maturation stage of collagen fibrils within the newly formed bone matrix, where birefringence brightness intensity is directly related to the maturation process. To assess collagen maturation, varying birefringence patterns—green, yellow, and red—were observed in the affected regions. Thicker, more anisotropic collagen fibers exhibited birefringence in hues ranging from orange to red, while thinner, disorganized fibers displayed shades of yellow to green. In groups undergoing photobiomodulation treatment (PBFG, PFG, PCG), qualitative findings indicated improved organization and maturation of collagen fibers, characterized by predominant orange-red birefringence and a reduced presence of fibers with green-spectrum birefringence, suggesting a decrease in immature, thinner fibers (Figure 5).

Over the 14-day period, histological images of PRS revealed a predominance of colors associated with thin and disorganized collagen fibers in the bone neoformation area, characteristic of immature bones. The presence of yellowish-green birefringence was minimal and sporadic. By day 42, a notable increase in these yellowish-green hues was observed, indicating the progression of bone maturation in the neoformation regions. The groups showing the highest presence of yellowish-green birefringence were those treated with trans surgical low-level laser (PBFG, PFG, PCG), as well as the group that received fibrin biopolymer (FG) (Figure 5).

### 3.3 Immunohistochemistry analysis

**FIGURE 6:**
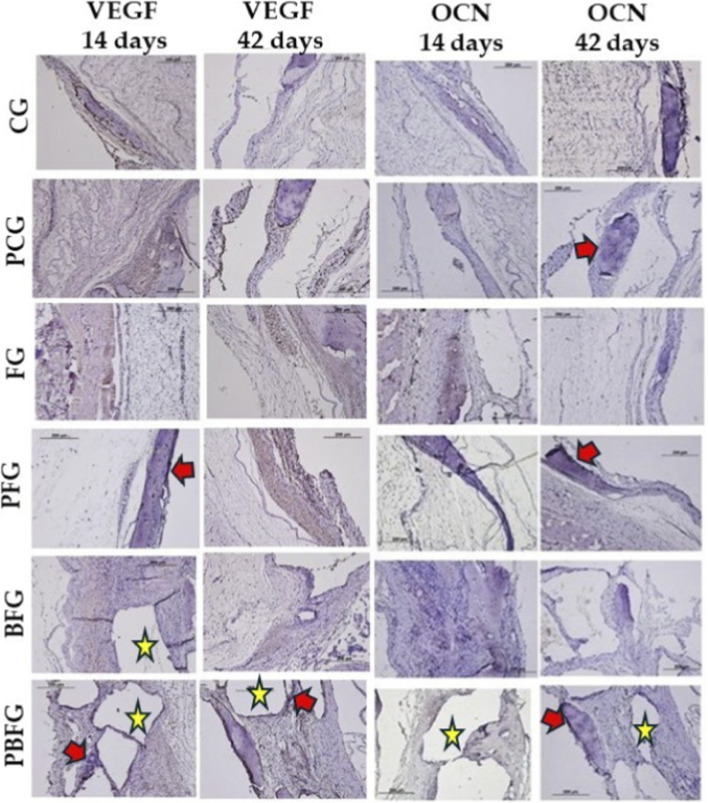
Immunohistochemistry for VEGF (top) and OCN (bottom) in 8 mm calvarial defects at 14/42 days (20×). CG (clot), PCG (PBM + clot), FG (fibrin), PFG (PBM + fibrin), BFG (β-TCP + fibrin), PBFG (PBM+β-TCP + fibrin). Yellow stars: β-TCP particles. Red arrows: positive immunostaining. Scale bar: 200 μm. Enhanced VEGF (FG/PFG); OCN around β-TCP (BFG/PBFG).

**FIGURE 7:**
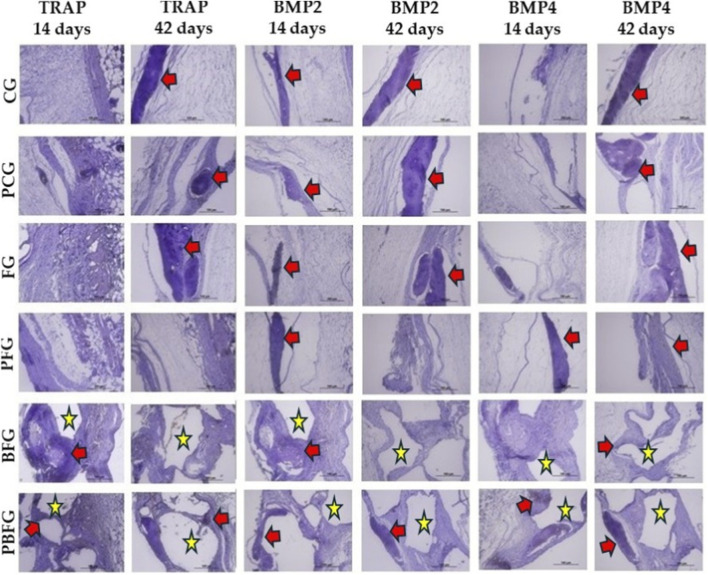
Immunohistochemistry for TRAP, BMP2, and BMP4 in 8 mm calvarial defects at 14/42 days (20×). CG (clot), PCG (PBM + clot), FG (fibrin), PFG (PBM + fibrin), BFG (β-TCP + fibrin), PBFG (PBM+β-TCP + fibrin). Yellow stars: β-TCP particles. Red arrows: positive immunostaining. Scale bar: 200 μm. Prominent BMP expression in PFG/PBFG.

The immunohistochemical technique used to detect VEGF, OCN, TRAP, BMP2, and BMP4 showed high specificity in detecting these proteins, which was confirmed by the total absence of staining in the negative control. In the VEGF immunostaining, positive staining for angiogenic growth factor was observed in all groups, being more pronounced in the groups with the presence of fibrin biopolymer (FG and PFG) at 14 and 42 days. In the OCN immunostaining, positive staining for this transcription factor was observed in the tissue and around the biomaterial particles of the BFG and PBFG groups at 14 and 42 days (Figures 6, 7).

### 3.4 Statistical analysis

**FIGURE 8:**
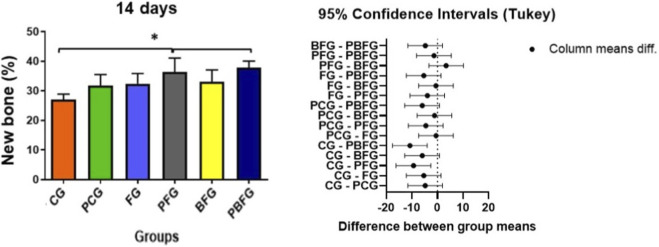
Means and standard deviations of the percentage of newly formed bone tissue after a 14-day experimental period. Asterisk (*) indicates significant differences between groups (*p* ≤ 0.05). CG (Clot group); PCG (PBM + Clot); FG (Fibrin group); PFG (PBM + FG); BFG (Biomaterial group + Fibrin); PBFG (PBM + Biomaterial + F).

In the 14-day period, there was a significant difference between the CG in relation to the PFG and PBFG groups (*p* ≤ 0.05). In the comparative analysis between the other groups, there was no significant difference (Table 1; Figure 8).

**TABLE 1:** Percentage (%) of new bone tissue formed in the 14-day experimental period.

14 days	CG	PCG	FG	PFG	BFG	PBFG
Percentage (%) of new bone formed	27.0 ± 1.87*	31.8 ± 3.7	32.4 ± 3.43	36.4 ± 4.67^a^	33.0 ± 4.06	37.8 ± 2.28^a^

ANOVA with Tukey post-test (*p* ≤ 0.05). Values expressed as mean and standard deviation. CG (Clot group); PCG (PBM + Clot); FG (Fibrin group); PFG (PBM + FG); BFG (Biomaterial group + Fibrin); PBFG (PBM + Biomaterial + F).

^a^ Significant difference.

At 14 days, one-way ANOVA revealed a significant effect of treatment on the percentage of newly formed bone (F(5,24) = 5.99, *p* = 0.001), with a large effect size (η^2^ = 0.56), indicating that approximately 56% of the variance in early bone formation was attributable to the different filling materials and PBM protocols. Groups treated with PBM in combination with fibrin (PFG) or with the full biocomplex (PBFG) exhibited higher percentages of newly formed bone compared with the clot control: PFG showed 36.4% ± 4.67% *versus* 27.0% ± 1.87% in CG, with a very large effect size (Cohen’s *d* = 2.64), whereas PBFG reached 37.8% ± 2.28% compared with 27.0% ± 1.87% in CG, yielding an extremely large effect size (Cohen’s d = 5.18).

**FIGURE 9:**
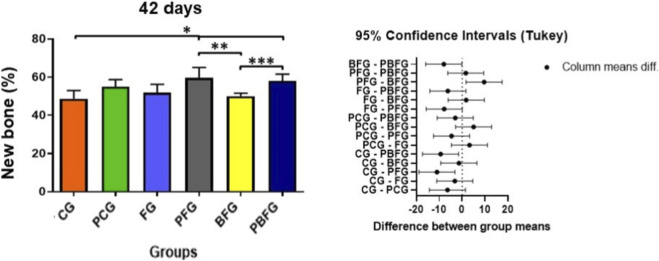
Means and standard deviations of the percentage of newly formed bone tissue after a 42-day experimental period. Asterisks (*, ** and ***) indicate significant difference between groups (*p* ≤ 0.05). CG (Clot group); PCG (PBM + Clot); FG (Fibrin group); PFG (PBM + FG); BFG (Biomaterial group + Fibrin); PBFG (PBM + Biomaterial + F).

In the 42-day period, there was a significant difference between the CG in relation to the PFG and PBFG, as well as between the PFG vs. BFG and BFG vs. PBFG (*p* ≤ 0.05). In the comparative analysis between the other groups, there was no significant difference (Table 2; Figure 9).

**TABLE 2:** Means and standard deviations of the percentage (%) of new bone tissue formed in the experimental period of 42 days.

42 days	CG	PCG	FG	PFG	BFG	PBFG
Percentage (%) of new bone formed	48.6 ± 4.34*	55.0 ± 3.67	51.8 ± 4.44	59.6 ± 5.5*^,^**	50.1 ± 1.58**^,^***	58.1 ± 3.54*^,^ ***

ANOVA with Tukey post-test (*p* ≤ 0.05). Asterisks (*, ** and ***) indicate a significant difference between the groups. Values expressed as mean and standard deviation. CG (Clot group); PCG (PBM + Clot); FG (Fibrin group); PFG (PBM + FG); BFG (Biomaterial group + Fibrin); PBFG (PBM + Biomaterial + F).

At 42 days, the treatment effect on new bone formation remained significant (F (5,24) = 6.06, *p* = 0.0009), with a large effect size (η^2^ = 0.56), demonstrating that roughly 56% of the variance in late bone regeneration was explained by the treatment modalities. PFG presented 59.6% ± 5.50% of newly formed bone compared with 48.6% ± 4.34% in CG (Cohen’s *d* = 2.22), while PBFG reached 58.1% ± 3.54% *versus* 48.6% ± 4.34% in CG (Cohen’s *d* = 2.40); additionally, PBFG showed higher new bone formation than BFG (58.1% ± 3.54% vs. 50.1% ± 1.58%; Cohen’s *d* = 2.92), underscoring the substantial incremental benefit of associating PBM with β-TCP and fibrin in critical calvarial defects.

**FIGURE 10:**
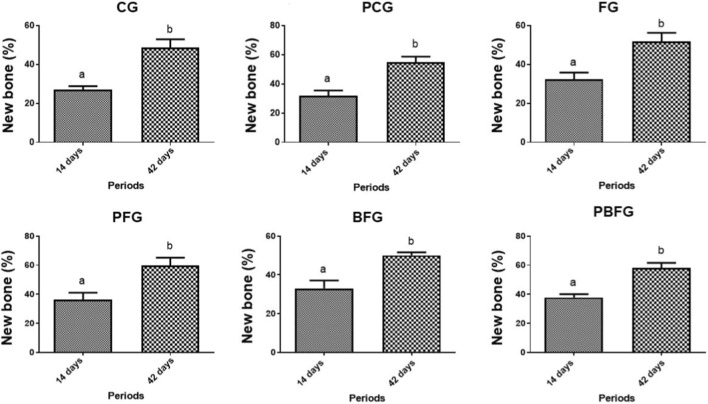
Temporal evolution of new bone formation (mean ± SD) across groups at 14 vs. 42 days. Different lowercase letters (a,b) indicate significant intragroup differences (*p* < 0.05; Student’s t-test). CG (clot), PCG (PBM + clot), FG (fibrin), PFG (PBM + fibrin), BFG (β-TCP + fibrin), PBFG (PBM+β-TCP + fibrin).

When comparing the experimental periods, 14 vs. 42 days, in each of the groups, there was a significant difference between the two periods in all groups (Figure 10).

## 4 Discussion

The purpose of this preclinical research was to assess the effectiveness of a novel biocomplex combining tricalcium phosphate-based synthetic biomaterial, a new heterologous fibrin biopolymer formulation, and an intraoperative photobiomodulation protocol for treating critical bone defects. The association of fibrin biopolymer with the transoperative photobiomodulation protocol was effective, improving the process of new bone formation.

Fourteen days after surgery, micro-CT images revealed progress in bone repair, especially in the CG and PFG groups. In the BFG and PBFG groups, the radiopaque biomaterial hindered clear visualization of new bone formation, although the defect cavity remained qualitatively observable. Bone formation occurred primarily at the edges of the defect, as evidenced by similar studies (Santos et al., 2015; Tu et al., 2022). Traditional approaches for assessing and measuring bone growth in calcium phosphate (CaP) scaffolds using micro-CT imaging often present limitations, highlighting the need for standardized evaluation protocols to ensure consistency and comparability across studies. While the research conducted by Lewin et al. (2017) marked a step forward, the authors emphasized the ongoing need to refine segmentation techniques for tabletop micro-CT images to more accurately quantify bone formation within CaP scaffolds (Lewin et al., 2017).

At 42 days, micro-CT images show more advanced bone repair, especially in CG and PFB groups, with a better-organized structure than at 14 days. Although distinguishing new bone from biomaterial in BFG and PBFG groups remains difficult, increased mineralization allows observation of bone formation around the biomaterial. The result we found is typical of repair processes with biocompatible materials, providing tissue organization in more advanced periods of post-operative time (Liang et al., 2021; Otto et al., 2021; Bu et al., 2024). The bone repair process increases radiopacity, due to an increase in the amount of calcium (Bilvinaite et al., 2022).

β-TCP remnants positively modulated biomineralization through controlled degradation, releasing Ca^2+^ and PO_4_^3-^ ions that created a supersaturated microenvironment conducive to hydroxyapatite nucleation and matrix mineralization. This osteoconductive mechanism was evidenced by upregulated OCN immunostaining surrounding particles (Figure 6), enhanced BMP2/4 expression in PBFG/PFG groups, and histomorphometric demonstration of 19.5% greater bone volume in PBFG *versus* CG (58.1% ± 3.54% vs. 48.6% ± 4.34%; *p* < 0.05), despite micro-CT quantification limitations due to biomaterial radiopacity (Garcia et al., 2022).

Histomorphologically, within a period of 14 days, the PBFG, PFG and PCG groups (treated with PBM) showed new bone formation. In groups with biomaterial (BFG and PBFG), reactive tissue and limited bone formation appeared between particles in a centripetal pattern, mainly at the edges of the defect. In groups with only fibrin biopolymer (FG and PFG), it was seen as hyperchromatic bodies within connective tissue, accompanied by mild inflammation. The distinction between mature and primary bone was clear across all groups. Reactive connective tissue presents a mild inflammatory reaction, typical of the initial phases of bone repair, with no evidence of foreign body reaction to the grafted biomaterials, as demonstrated in studies involving ceramics and fibrin derivatives (Batool et al., 2021; Reis et al., 2022; Amani et al., 2024). Laser therapy (PBM) shows a milder response and local neovascularization, important for incorporation or integration of grafts (De Marco et al., 2022; Klassmann et al., 2023).

At 42 days, bone growth advanced, though mature and immature areas blurred at wound edges. BFG and PBFG showed stronger centripetal formation, with some biomaterial fully encased. Angiogenesis increased in all fibrin groups, especially with photobiomodulation. In rats, non-critical bone defects are completely repaired in approximately 6 weeks (Vicentis et al., 2025). In our study, we used critical 8 mm defects in the calvaria, which do not repair until the end of the experiment. In this way, we can evaluate the performance of isolated or combined grafts, in addition to PBM, and it was possible to observe histological results compatible with previous studies by our group (Rossi et al., 2024) or other researchers (Costa De-Moraes et al., 2025), of more organized bone and integration with ceramic particles (Yoshikawa et al., 2009). The fibrin biopolymer was not evidenced in this period (Karp et al., 2004; Noori et al., 2017; Bighetti et al., 2024).

Histochemical analysis of collagen fibers (Picrosirius-red staining, PRS) revealed that birefringence intensity correlates with fibril maturation. Orange-red hues indicated thicker, organized fibers, while green-yellow suggested immature, disorganized ones. In PBM-treated groups (PBFG, PFG, PCG), collagen showed improved maturation and organization, with more orange-red and fewer green-spectrum fibers. PRS, when paired with polarized light microscopy (PLM), effectively highlights collagen fibers due to their birefringence, offering superior clarity over conventional stains. PLM further reveals fiber orientation, classification, and distribution, essential for evaluating collagen changes. This affordable and straightforward method effectively identifies collagen fibers and maps their distribution, clearly distinguishing type I collagen from the green-stained type III (Gerbi et al., 2018; Liu et al., 2021).

The immunohistochemical method effectively detected VEGF, OCN, TRAP, BMP2, and BMP4, with no staining in negative controls confirming its specificity. VEGF staining was observed in all groups, especially in FG and PFG on 14 and 42 days. OCN expression appeared in tissues and around biomaterial in BFG and PBFG at the same time points. VEGF was analyzed due to its central role in angiogenesis—the formation of new blood vessels—which is vital for bone healing (Grosso et al., 2023). In cases of bone damage or defects, sufficient vascularization is necessary to deliver oxygen, nutrients, and reparative cells to the affected area (Shineh et al., 2023). Low-level laser therapy (LLLT), also known as photobiomodulation (PBM), has been investigated for its ability to enhance VEGF expression, which may boost blood vessel formation and speed up both healing and bone regeneration (Berni et al., 2023; Tamimi et al., 2025).

Osteocalcin (OCN) serves as a reliable indicator of osteoblast function, signaling that bone matrix mineralization is actively occurring—a critical factor for evaluating the progression and quality of bone regeneration (Indarwulan et al., 2024). In addition, OCN ranks among the most prevalent non-collagenous proteins in bone and plays a key role in the maturation of bone tissue (Mergoni et al., 2016; Lee et al., 2019; Wang et al., 2024). OCN immunostaining may represent an osteoblastic attraction that occurred due to the action of ceramics and fibrin biopolymer, favoring cell migration and proliferation due to its three-dimensional structural characteristics and porosity, essential in the recomposition of injured tissues (Wang et al., 2023).

BMP2 and BMP4, members of the TGF-β family, signal the initiation of osteogenic activity, reflecting new bone formation. They play key roles in driving stem cells to differentiate into osteoblasts, enhancing bone matrix mineralization, and boosting overall bone-forming processes. Representative labeling by BMPs was evidenced in this study, mainly in PFG and PBFG, groups that contained fibrin and PBM in both. Their association has been previously studied, with promising results (de Oliveira Gonçalves et al., 2016; Reis et al., 2022), in lesions of bone tissue or others, such as nerves (Cunha X. et al., 2021; Rodrigo et al., 2024) and skin (Medrado et al., 2008; Silva et al., 2023). TRAP marking, especially at 14 days, indicates the presence of the bone remodeling or degradation process, which is consistent with research on the same topic (Saito et al., 2011; Schini et al., 2023).

Quantitatively, after statistical analysis, it was observed that in the 14-day period there was a significant difference between the CG in relation to the PFG and PBFG groups (*p* ≤ 0.05). In the 42-day period, there was a significant difference between the CG in relation to the PFG and PBFG, as well as between the PFG vs. BFG and BFG vs. PBFG (*p* ≤ 0.05). Therefore, PBM with LLLT was a factor that contributed to increasing the percentage of new bone formed, regardless of the type of treatment used. The association of fibrin biopolymer favors insertion into the cavity, hemostasis and forms an active biocomplex (Buchaim et al., 2022; Reis et al., 2022), providing an ideal microenvironment for cell growth and angiogenic factors (Huang et al., 2018). Furthermore, it can be hypothesized that the smaller amount of fibrinogen that we used in this study, in the composition of FB, may have formed a less dense fibrin network (Kim et al., 2014; Vasconcelos et al., 2016; Abas et al., 2022), which was necessary for the initial use of FB as peripheral nerve glue (Buchaim et al., 2015).

Therefore, photobiomodulation (PBM) administered *via* an 808 nm infrared laser (100 mW, 1.016 W/cm^2^, 60.98 J/cm^2^ per point), used in this preclinical experimental protocol, presents a main mechanism involving the absorption of photons by cytochrome c oxidase in mitochondrial membranes. This photonic stimulation increases ATP generation, alters membrane dynamics, and facilitates calcium influx, collectively shaping essential biochemical pathways in bone tissue (Dong et al., 2022). These combined effects mitigate inflammatory responses, trigger angiogenic activity, and expedite bone repair, corroborated by pronounced orange-red birefringence in PRS and elevated immunohistochemical signals in PBM-treated specimens. For critical calvarial defects, the protocol adopted in this study enhances the microenvironment established by the heterologous fibrin biopolymer and β-TCP, promoting Ca/PO_4_ ion liberation that drives hydroxyapatite nucleation and supports a higher yield of new bone formation (Rodrigues Júnior et al., 2025).

Therefore, the use of combined therapies in this study can be envisaged as clinical perspectives. Fibrin biopolymer should enter phase III of clinical studies soon, with its phase II completed for use in chronic venous ulcers (Abbade et al., 2020). PBM is already reality, but its protocols are diverse and not standardized. Ceramics are constantly being tested and marketed, as is the case in this study with QualyBone TCP (QualyLive^®^, Amadora, Portugal).

A primary limitation of this study is the absence of quantitative biomechanical characterization of the newly formed bone tissue, which precludes direct assessment of its mechanical quality and functional competence. While histomorphometric analysis demonstrated substantial increases in bone volume (η^2^ = 0.56; Cohen’s *d* = 2.22–5.18), the quantity of regenerated tissue does not necessarily correlate with its biomechanical integrity (Karpiński et al., 2022), as emphasized in recent investigations of β-TCP admixtures in bone cements where compressive strength varied significantly with ceramic concentration (>2% β-TCP maintained favorable properties up to 10% admixture) (Szabelski et al., 2024a, 2024b). Similarly, micro-CT quantification was inherently limited by the radiopacity of QualyBone® β-TCP particles (0.5–1.0 mm), which obscured precise volumetric assessment of new bone formation in BFG and PBFG groups, consistent with established challenges in calcium phosphate scaffold imaging that necessitate complementary histological validation. Future studies should incorporate nanoindentation, three-point bending, or micro-computed tomography with dual-energy scanning to evaluate elastic modulus, ultimate strength, and fracture toughness of the regenerated bone-biomaterial interface.

## 5 Conclusion

This *in vivo* study aimed to evaluate an intraoperative photobiomodulation protocol combined with a novel biocomplex composed of synthetic tricalcium phosphate ceramic (99.9% β-TCP) and a heterologous fibrin biopolymer in critical calvarial defects in rats. Single-session intraoperative photobiomodulation (PBM) significantly promoted new bone formation within the surgical cavity, with the PBFG group exhibiting a 19.5% increase compared to the CG group (58.1 ± 3.54 vs. 48.6 ± 4.34). This enhanced bone regeneration was primarily associated with the heterologous fibrin biopolymer formulation, which utilized a lower fibrinogen concentration relative to previous bone repair studies. The results of the associations of PBM, FB and β-TCP are promising and have translational potential, after a phase III clinical study of FB, which can be used clinically.

## Data Availability

The original contributions presented in the study are included in the article/supplementary material, further inquiries can be directed to the corresponding author.
